# Efficiency of Commercial Egg Yolk-Free and Egg Yolk-Supplemented Tris-Based Extenders for Dromedary Camel Semen Cryopreservation

**DOI:** 10.3390/ani9110999

**Published:** 2019-11-19

**Authors:** Ayman Abdel-Aziz Swelum, Islam M. Saadeldin, Hani Ba-Awadh, Mohsen G. Al-Mutary, Abdullah F. Moumen, Abdullah N. Alowaimer, Hany Abdalla

**Affiliations:** 1 Department of Animal Production, College of Food and Agriculture Sciences, King Saud University, P.O. Box 2460, Riyadh 11451, Saudi Arabia; isaadeldin@ksu.edu.sa (I.M.S.); vet.hani77@gmail.com (H.B.-A.); moumen.2173@gmail.com (A.F.M.); aowaimer@ksu.edu.sa (A.N.A.); 2 Department of Theriogenology, Faculty of Veterinary Medicine, Zagazig University, 44519 Zagazig, Egypt; lotfi_hany@yahoo.com; 3 Department of Physiology, Faculty of Veterinary Medicine, Zagazig University, 44519 Zagazig, Egypt; 4 Department of Basic Sciences, College of Education, Imam Abdulrahman Bin Faisal University, Dammam 31451, Saudi Arabia; mgalmutary@iau.edu.sa

**Keywords:** semen, Triladyl, Steridyl, AndroMed, OPTIXcell, SHOTOR

## Abstract

**Simple Summary:**

This study compared the efficiency of commercial egg yolk-free (AndroMed, OPTIXcell) and egg yolk-supplemented (Triladyl, Steridyl) Tris-based extenders for semen cryopreservation in dromedary camels. The camel-specific extender SHOTOR was used for reference. SHOTOR, Triladyl, Steridyl, AndroMed, and OPTIXcell can all be used for camel semen cryopreservation; however, SHOTOR and Triladyl provide the best post-thawing sperm quality.

**Abstract:**

This study compared the efficiency of commercial egg yolk-free (AndroMed, OPTIXcell) and egg yolk-supplemented (Triladyl, Steridyl) Tris-based extenders for semen cryopreservation in seven adult dromedary camels. The camel-specific extender SHOTOR was used as control. The collected semen samples were evaluated and diluted with SHOTOR, Triladyl, Steridyl, AndroMed, or OPTIXcell. The diluted semen was gradually cooled and equilibrated for two hours before liquid nitrogen freezing. Semen was evaluated prior to freezing and after freeze-thawing cycles for motility, kinetics, vitality, abnormality, plasma membrane integrity, and DNA fragmentation using computer-assisted sperm analysis. In pre-freezing evaluation, progressive sperm motility was higher in SHOTOR-diluted samples (21.54 ± 1.83) than in samples diluted with Steridyl, OPTIXcell, or AndroMed (15.76 ± 1.80, 17.43 ± 1.10, and 13.27 ± 1.07, respectively). Moreover, Triladyl and SHOTOR resulted in significantly (*p* < 0.05) better sperm vitality and DNA integrity than all other diluents, but Triladyl resulted in a significantly (*p* < 0.05) better plasma membrane integrity (87.77 ± 0.31) than SHOTOR (85.48 ± 0.58). In the post-thawing evaluation, Triladyl led to significantly (*p* < 0.05) higher sperm motility (38.63 ± 0.81%; *p* < 0.05) when compared to SHOTOR, Steridyl or AndroMed (35.09 ± 1.341%, 34.4 ± 0.84%, and 31.99 ± 1.48%, respectively), with OPTIXcell being the least efficient (28.39 ± 0.86%). Progressive sperm motility was the highest when using Triladyl. Post-thawing curvilinear, straight line and average path sperm velocities were highest with Triladyl and lowest with AndroMed. Triladyl led to the highest linearity coefficient and straightness sperm coefficient, while SHOTOR to the highest DNA and plasma membrane integrity. OPTIXcell and AndroMed resulted in poor post-thawing sperm vitality, while Steridyl was less efficient than Triladyl. The highest rate of sperm abnormalities was recorded with OPTIXcell and the lowest with SHOTOR or Triladyl. In conclusion, SHOTOR, Triladyl, Steridyl, AndroMed, and OPTIXcell can all be used for camel semen cryopreservation; however, SHOTOR and Triladyl provided the best post-thawing sperm quality. Based on our findings, Triladyl is the best commercially available extender for dromedary camel semen cryopreservation to date.

## 1. Introduction

Artificial insemination (AI) has introduced numerous improvements in the livestock industry particularly with the possibility for selection and improvement of genetic-associated traits [1]. Semen cryopreservation is an essential requirement for the widespread application of AI as it overcomes time- and space-related limitations. In bovine, the application of AI has grown considerably with a worldwide semen trade having been developed [2]. To date, there are multiple challenges associated with different steps of AI in camelids, including semen collection, handling, and preservation [3,4]. 

Semen extenders are liquid diluent buffers that contain protective substances. They determine the efficiency of cryopreservation protocols and thus play a key role in semen protection [5]. Therefore, numerous efforts have been made to define the best semen extender in different animal species including Camelidae [6,7,8]. These efforts ended with the formulation of many commercial semen extenders for bovine and equine, while for camelids there are mainly two specific semen extenders SHOTOR and Green buffer. SHOTOR has proven successful in the preservation of both chilled and frozen Bactrian camel semen [9,10] as well as dromedary camel semen [6]. Moreover, Green buffer has proven successful in the preservation of frozen dromedary camel semen [7]. However, these extenders have not shown a reasonable in vivo fertility rates and their success is based mainly on post-thawing evaluation. Green buffer is only commercially produced dromedary camel semen extender, but its availability is limited which might be due to the limited application of AI in camelids and the relatively smaller population size, compared to other species. Most AI centers prefer to use commercial extenders because of their efficacy, quality, hygienic production, safety, and easy preparation. The limited availability of camel-specific commercial diluent leading to utilization of other species diluent for preservation of camel semen, for example, INRA-96 for freezing camel semen [7] and Triladyl, AndroMed as well as OPTIXcell for chilled camel semen [11,12]. 

The most common ingredients added to extenders when used for freezing semen of different species, including camelids are Tris buffer and egg yolk [6,10,13,14,15,16,17,18,19]. However, the use of egg yolk in extenders has been proven problematic for semen cryoprotection, leading to the search for potential animal-free substitutes [5]. The most commonly used substitutes are soy lecithin, low-density lipoprotein (LDL), and liposomes [5,20]. Promising results have been published regarding the use of animal-free egg substitutes for semen cryopreservation [5,20,21,22]. AndroMed and OPTIXcell are the most common bull extenders that are supplied with egg yolk replacer. AndroMed contains soy lecithin while OPTIXcell contains liposomes. Despite being developed as bovine-specific, they have led to efficient semen cryoprotection in other species including, rams, cats, and Iberian red deer [12,23,24,25,26]. AndroMed as well as OPTIXcell have been tried for preservation of camel semen in chilled condition [11,12]. To the best of our knowledge, no previous reports have investigated the efficiency of egg yolk replacers in the cryopreservation of dromedary camel semen. Therefore, the present study compared the cryopreservation efficiency of egg yolk-free (AndroMed, OPTIXcell) and egg yolk-supplemented (Triladyl, Steridyl) commercial extenders, as well as of the in-lab formulation SHOTOR, for dromedary camel semen.

## 2. Materials and Methods 

### 2.1. Animals and Experimental Design

Seven adult, fertile, dromedary camel bulls between 6–7 years of age and a mean body condition score of 3 (0-extremely thin, 5-obese) were employed in the current study. Camel bulls were housed individually at an experimental farm in the Department of Animal Production, King Saud University, Riyadh, Saudi Arabia. Animals were fed a combination of alfalfa hay, straw, and commercial pellets (ARASCO, Riyadh, Saudi Arabia), with free access to fresh water. In the current experiment, the animals were not subjected to any harm or suffering and did not receive any experimental treatment. Hence, ethical approval from the institutional ethical committee was not required. 

### 2.2. Chemicals and Extenders Preparation

The chemicals used for the preparation of the lab-formulated semen extender SHOTOR include, Tris (Merck, Darmstadt, Germany), citric acid (Winlab, Market Harborough, UK), glucose (Sigma, Saint Louis, US), fructose (Sigma, Saint Louis, US), gentamicin (Sigma, Saint Louis, US), and glycerol (Sigma, US). The commercially used semen extenders were Triladyl (Minitube, Tiefenbach, Germany), Steridyl (Minitube, Tiefenbach, Germany), AndroMed (Minitube, Tiefenbach, Germany), and OPTIXcell (Imv technologies, L’Aigle, France). Triladyl contains Tris, citric acid, sugar, buffers, glycerol, water, and antibiotics (tylosin, gentamicin, spectinomycin, lincomycin). Steridyl contains Tris, citric acid, sugar (fructose), buffers, glycerol, water, irradiated sterile egg yolk, and antibiotics (tylosin, gentamicin, spectinomycin, lincomycin). The formulation of AndroMed includes phospholipids, Tris, citric acid, sugar (fructose), antioxidants, buffers, glycerol, water, and antibiotics (tylosin, gentamicin, spectinomycin, lincomycin). OPTIXcell contains carbohydrates, mineral salts, buffers, antioxidants, phospholipids (liposome-based formula and animal-free protein), water, glycerol, and antibiotics (tylosin, gentamicin, spectinomycin, lincomycin).

The lab-formulated semen extender SHOTOR consisted of 214.6 mM Tris, 64.2 mM citric acid, 66.6 mM glucose, and 49.9 mM fructose with 330 mOsm/kg osmolality and pH 6.9. SHOTOR was supplied with 20% freshly prepared egg yolk, 3% glycerol, and 0.1 mg/mL gentamicin. The commercially acquired diluents were prepared according to the manufacturer’s instructions. Briefly, Triladyl was prepared through a 1:3 dilution with bidistilled water and further supplementation with 20% v/v of freshly-prepared egg yolk. Steridyl was prepared through a 1:1.5 dilution with bidistilled water. AndroMed was prepared through a 1:4 dilution with bidistilled water and OPTIXcell via a 1:2 dilution with bidistilled water. 

### 2.3. Semen Processing and Cryopreservation 

During breeding season, ejaculates were collected using an artificial vagina set-up twice weekly, early in the morning. Ejaculates that showed normal volume (≥2 mL), normal color (milky white), and free from contamination were divided into five samples. Each sample was divided between five diluents and gradually diluted (1:4 to obtain about 120 × 10^6^ sperm/mL) in a warm water bath (37 °C) with one of the diluents under investigation and manually liquefied by repeated gentle pipetting for 45–90 min depending on its viscosity [27]. After liquefaction, initial sperm motility assessment was performed using a computer-assisted semen analysis (CASA) system (Sperm Class Analyzer; SCA, version 4.0.0.5, Microptic S.L., Barcelona, Spain). Ejaculates with total sperm motility less than 60% were excluded from the study. The diluted semen samples were transferred with a water jacket container to a refrigerator to achieve gradual cooling from 37 °C to 5 °C within 2 h. After a 2-h equilibration period at 5 °C, the semen samples were loaded into 0.25-mL straws. Just before freezing, representative samples were evaluated (pre-freezing evaluation). The straws were frozen for 10 min at 8 cm above the liquid nitrogen surface followed by immersion in liquid nitrogen. The frozen semen was stored for at least 48 h before completing the post-thawing evaluation. The experiment was repeated five times.

### 2.4. Semen Assessment

At least three straws per extender per repeat were separately evaluated after thawing. The straws were thawed at 37 °C for 30 s and sperm motility, vitality, morphology, plasma membrane integrity, and DNA fragmentation were evaluated.

#### 2.4.1. Sperm Motility 

Semen samples were diluted again by Tris buffer 1:4 or 1:5 to have sperm concentration about 25 × 10^6^ sperm/mL. Sperm motility was evaluated using Leja slides (20 micron–4 chambers) under a phase contrast microscope and a 20× objective with the use of CASA (sperm class analyzer—SCA, version 4.0.0.5, Microptic S.L., Barcelona, Spain). At least seven random microscopic field videos and 200 spermatozoa were evaluated. The image size was 768 × 576 pixels. The acquisition frame rate was set at 75 frames per second. The CASA output criteria included the proportion of motile sperm and the proportion of sperm showing progressive motility. Sperm motility kinetics were also evaluated. Sperm motion velocity was measured via three ways: (a) Average pathway velocity (VAP—the average smoothed sperm position); (b) straight-line velocity (VSL—the straight-line distance between the beginning and end of a sperm path); and (c) curvilinear velocity (VCL—the total distance travelled by a spermatozoon, including every point in the cell path divided by the time elapsed). The progressiveness indicators: (i) linearity (LIN = VSL/VCL) and (ii) straightness (STR = VSL/VAP) were calculated and expressed as a percentage. The amplitude of lateral head displacement (ALH—the maximum value of the distance of any point on the cell track from the average path multiplied by 2) and beat cross frequency (BCF—the frequency with which the cell track crosses the average cell path in either direction expressed in Hz) were recorded [28]. Based on the SCA settings, sperm motility was classified into various subpopulations. The SCA settings were as follows: Particle area: 3–70 μm^2^; VCL cut-off values: 10 µm/s < slow < 45 µm/s < medium < 75 µm/s < rapid; progressivity: > 80% of STR; circular: < 50 % LIN; connectivity: 12 pixels; VAP points: 5 pixels.

#### 2.4.2. Sperm Vitality 

Sperm vitality was assessed using a FluoVit kit (Microptic S.L., Barcelona, Spain) following the manufacturer’s instructions [29,30,31]. Briefly, 10 µL of semen was mixed with 1 µL of warm (37 °C) Hoechst trihydrochloride trihydrate stain. Five min later, 1 µL of warm (37 °C) propidium iodide was added and the mixture was incubated for five additional min. The sample was mounted on a standard slide with a cover glass and visualized via the fluorescent emission unit attached to the SCA system. Dead sperm was stained red while viable sperm blue. At least 200 spermatozoa were evaluated and the proportion of viable sperm was calculated.

#### 2.4.3. Sperm Plasma-Membrane Integrity

Spermatozoa plasma membrane integrity was assessed using the hyposmotic swelling (HOS) technique [3]. Briefly, 50 µL of semen was mixed with 200 µL of HOS solution (a fructose sodium citrate solution with ~60 mOsm/kg osmotic pressure) and incubated for 45 min at 35 °C. The sample was then transferred to a glass slide, covered with a cover slip, and examined under a light microscope (400×). Sperm with intact plasma membranes display coiled tails while those with disrupted membranes display straight tails. At least 200 spermatozoa were evaluated and the proportion of sperm with intact plasma membrane was calculated.

#### 2.4.4. Sperm DNA Fragmentation

Sperm DNA fragmentation was assessed with the Halomax DNA kit (Halotech, Madrid, Spain) following the manufacturer’s instructions [29,30,31]. Briefly, the agarose gel was incubated at 90–100 °C for 5 min followed by 5 min at 37 °C. Twenty-five microliter of semen was mixed with 50 µL of agarose gel. Twenty microliters of the mixture were then transferred to a super-coated slide and covered with a 22 × 22 mm coverslip. The slide was incubated at 4 °C for 5 min. The coverslip was removed carefully, and the slide was immersed into an acidic solution (80 μL HCl in 10 mL of distilled water) for 7 min. Thereafter, the slide was immersed in a lysing solution for 25 min. After rinsing with distilled water, the slide was dehydrated for 2 min in increasing ethanol concentrations (70%, 90%, and 100%). Finally, the slide was incubated with the Wright stain for 60 min, rinsed under tap-water, and allowed to dry at 25 °C. The slide was examined under a light microscope (100×). Sperm nuclei with fragmented DNA produce either a small halo or lack one altogether, but nuclei without DNA fragmentation produce big halos. The observed halos were examined and spermatozoa with large or medium halos were not considered negative for DNA fragmentation, while sperm with small, degenerated halos or with no halos were considered positive for DNA fragmentation. At least 200 spermatozoa were evaluated and the proportion of sperm with fragmented DNA was calculated.

#### 2.4.5. Sperm Morphology and Morphometric Evaluation

A routine semen smear was prepared and air dried. The slides were immersed in New Rapid SpermBlue fixative/stain (Microptic S.L., Barcelona, Spain) for 1–2 min. The excess stain was drained off, and the slides were dipped in distilled water for about 3 s. After drying, the stained smears were examined under a light microscope (600×). The morphology of minimum two hundred spermatozoa was examined, and total sperm abnormality was calculated manually and expressed as a percentage. Additionally, the shape of minimum 200 spermatozoa was morphometrically evaluated with SCA. The following distances were measured: (a) The distance along of the longitudinal axis in µm (L); (b) the distance along the small axis in µm (W); (c) the total area of the sperm head (LW) in µm2 (head area); (d) the area of the head occupied by the acrosome in % (acrosome); and (e) the sum of external boundaries in µm (head perimeter). After transferring the data into an excel spreadsheet, sperm ellipticity (L/W), rugosity (4pA/P2), elongation (L − W)/(L + W), and regularity (pLW/4A) were calculated. 

### 2.5. Statistical Analysis

For each sperm extender, at least fifteen straws were evaluated pre-freezing and post-thawing. The normality of data distribution was evaluated in the Shapiro–Wilk W test. Statistical differences among different types of extenders were evaluated by one-way analysis of variance (ANOVA) using a general linear model for all parameters with the statistical analysis system (SAS; SAS Institute, Cary, NC, US) to study the effect of cryopreservation and type of extenders. Significant differences between means were detected using Duncan’s multiple range test. Data were expressed as mean ± standard error (SE). Statistical significance was set at *p* < 0.05.

## 3. Results

The total number of ejaculates collected from 7 bulls for this study was 103 ejaculates. The average ejaculate volume was 5.74 ± 0.82 mL. Only 82 ejaculates passed the initial macroscopic criteria. The total number of samples passed the final criteria of initial microscopic evaluation was 70 ejaculates. These samples were equally distributed between different diluents. 

The sperm total motility, progressive motility, vitality, plasma membrane integrity, DNA integrity, and sperm abnormalities were significantly (*p* < 0.0001) retarded by freezing in all experimental diluents.

### 3.1. Effect of Different Extenders on Pre-Freezing and Post-Thawing Sperm Motility and Sperm Motility Kinetics

The effect of different extenders on pre-freezing and post-thawing sperm motility and sperm motility kinetics is presented in Table 1. Just before freezing, total sperm motility was similar (*p* = 0.1592) with all extenders and ranged from 78.29 ± 1.34 to 81.18 ± 0.65. Progressive sperm motility was higher (*p* < 0.0001) in SHOTOR-diluted samples (21.54 ± 1.83) than the ones diluted with Steridyl, OPTIXcell, or AndroMed (15.76 ± 1.80, 17.43 ± 1.10, and 13.27 ± 1.07, respectively). The proportion of rapid-progressive spermatozoa (class A) was significantly higher in SHOTOR-diluted samples when compared to any other diluent. The proportion of slow-progressive spermatozoa (class B) was significantly higher in semen samples diluted with SHOTOR than Steridyl or AndroMed, while the other diluents resulted in intermediate values. The proportion of non-progressive spermatozoa (class C) was significantly higher with AndroMed when compared with all other extenders, while SHOTOR resulted in the lowest proportion of non-progressive sperm than any other diluent.

Prior to freezing spermatozoa diluted with either Triladyl or OPTIXcell showed significantly faster VSL and VAP than sperm diluted with SHOTOR or AndroMed. Moreover, spermatozoa diluted with OPTIXcell showed a significantly (*p* < 0.0001) faster VCL than those diluted in SHOTOR, Steridyl, or AndroMed. The STR coefficient was significantly (*p* < 0.0001) greater when using Triladyl or OPTIXcell than with AndroMed or Steridyl, while it was at an intermediate value with SHOTOR. The LIN coefficient was higher with Triladyl, OPTIXcell, or AndroMed than SHOTOR or Steridyl. Sperm diluted with OPTIXcell showed significantly (*p* < 0.0001) higher BCF than that diluted in SHOTOR or Steridyl, while sperm diluted in any other diluent reached intermediate values. When using AndroMed or Triladyl as a diluent, sperm ALH was less than when diluting samples in any of other diluent. 

In the post-thawing evaluation, Triladyl resulted in significantly (*p* < 0.0001) higher total sperm motility (38.63 ± 0.81) than SHOTOR, Steridyl, or AndroMed (35.09 ± 1.341, 34.4 ± 0.84, and 31.99 ± 1.48, respectively), and the later three diluents resulted in significantly higher total motility than OPTIXcell (28.39 ± 0.86). Progressive sperm motility after using Triladyl (10.04 ± 0.66) was significantly (*p* < 0.0001) higher than with any other diluent (3.78 ± 0.32 to 5.13 ± 0.55). Moreover, Triladyl resulted in significantly more rapid-progressive spermatozoa than OPTIXcell and more slow-progressive spermatozoa than any other diluent. Ejaculates frozen in OPTIXcell resulted in significantly lower non-progressive sperm motility than those frozen in the other diluents. 

In post-thawing samples, sperm VAP, VSL, and VCL with Triladyl or AndroMed were significantly (*p* < 0.0001) faster than any other diluent; Steridyl showed significantly faster VAP, VSL, and VCL than SHOTOR, OPTIXcell, or AndroMed. Camel sperm frozen in Triladyl resulted in significantly (*p* < 0.0001) higher LIN and STR than that frozen in other diluents. Furthermore, Steridyl resulted in significantly lower LIN and STR than AndroMed. Triladyl and Steridyl resulted in significantly higher BCF than any other diluent. Sperm ALH values were significantly (*p* < 0.0001) variable among diluents with the highest and the lowest value reported with Triladyl and AndroMed, respectively. 

### 3.2. Effect of Different Extenders on Pre-Freezing and Post-Thawing Sperm Vitality, Plasma Membrane Integrity, DNA Integrity, and Sperm Abnormalities

The effect of different extenders on pre-freezing and post-thawing sperm vitality, plasma membrane integrity, DNA integrity, and sperm abnormalities is presented in Table 2. In the pre-freezing semen evaluation, Triladyl and SHOTOR resulted in significantly (*p* < 0.0001) better sperm vitality and DNA integrity than all other diluents, while Triladyl also led to a significantly (*p* < 0.0001) higher proportion of plasma membrane integrity (87.77 ± 0.31) than SHOTOR (85.48 ± 0.58). Steridyl resulted in the worst sperm vitality, integrity of plasma membrane, and DNA integrity. The highest percentage of sperm abnormalities was recorded with OPTIXcell (12.00 ± 0.06) and the lowest with SHOTOR and Triladyl (6.25 ± 0.08 and 6.50 ± 0.19, respectively).

In post-thawing semen samples, SHOTOR resulted in higher (*p* < 0.0001) sperm vitality, DNA integrity, and plasma membrane integrity than any other diluent. OPTIXcell and AndroMed resulted in the worst sperm parameters, while Steridyl resulted in significantly (*p* < 0.0001) lower values than Triladyl. The highest level of sperm abnormities was recoded with OPTIXcell and the lowest with SHOTOR or Triladyl. 

### 3.3. Effect of Different Extenders on Pre-Freezing and Post-Thawing Sperm Morphometry

The effect of different extenders on pre-freezing and post-thawing sperm head morphometry is presented in Table 3. In the pre-thawing evaluation, sperm head area and perimeter significantly (*p* < 0.0001) varied among samples with different diluents. Sperm elongation and ellipticity were significantly lower (*p* < 0.0001) when ejaculates were diluted and equilibrated with Triladyl or AndroMed. While, rugosity was significantly lower when ejaculates were diluted and equilibrated with OPTIXcell.

In the post-thawing evaluation, some sperm head morphometric parameters showed significant (*p* < 0.05) variation among samples with different diluents. Freezing of camel sperm in AndroMed resulted in a significantly shorter head perimeter than OPTIXcell and Triladyl. AndroMed resulted in a significantly lower ellipticity (*p* = 0.0117) and elongation (*p* = 0.0080) than other diluents except Steridyl. While, Steridyl resulted in significantly (*p* = 0.0124) low percentages of sperm regularity than SHOTOR and Triladyl. The percentage of the head area occupied by the acrosome was not significantly affected by type of diluent at pre-freezing (*p* = 0.3519) and post-thawing (*p* = 0.8985) evaluation.

## 4. Discussion 

The current study compared five semen extenders (four commercially available bovine extenders and one lab-formulated camel-specific extender). The precise composition of the commercial semen extenders is unknown and unpublished. Nevertheless, it is common knowledge that the main constituents of most diluents assessed in this study are Tris, citric acid, sugars, and glycerol. Previous studies have shown that the type of sugar, as well as sugar and citric acid concentration in Tris-based diluents significantly affect the efficiency of semen cryopreservation in camels [8,9,32]. Two of the extenders contain animal-free protein-like components as non-penetrating cryoprotectants (liposomes [OPTIXcell] or soy lecithin [AndroMed]) instead of egg yolk, while the remaining three contain egg yolk (Triladyl, Steridyl, SHOTOR). The overall results indicate that Triladyl resulted in significantly higher post-thawing total sperm motility and progressive sperm motility than any other diluent, while SHOTOR resulted in significantly better post-thawing sperm vitality, plasma membrane integrity, as well as DNA integrity than any other diluent. Furthermore, egg yolk replacer-supplied extenders resulted in lower post-thawing semen quality than egg yolk-based extenders. 

In the current study, post-thawing sperm vitality, plasma membrane integrity, DNA integrity, and motility were significantly lower with liposome- or soy lecithin-based extenders (OPTIXcell and AndroMed, respectively) than with any of the egg yolk-based extenders. In bovine, OPTIXcell resulted in significantly higher sperm motility, decreased acrosome reactions, and higher calving rates than egg yolk Tris-based extenders (Triladyl, Optidyl and homemade Tris-egg yolk) [20] as well as significantly higher bull sperm post-thawing quality than the egg yolk-based diluent Triladyl or other egg yolk-free commercial extenders (AndroMed, BioXcell) [33]. Furthermore, the efficiency of the soy lecithin-based extender BioXcell and Triladyl has been shown to be comparable [13], but BioXcell resulted in lowest total bull sperm motility, as well as plasma membrane and acrosome integrity, when compared to the egg yolk-supplied diluent Botu-Bov [34]. In buffaloes, OPTIXcell resulted in providing a better semen cryoprotection than soy lecithin- or egg yolk-based extenders [35,36], while AndroMed resulted in similar post-thawing sperm motility but better vitality and DNA integrity than egg yolk-based diluents [22]. Collectively the results of previous studies in cows and buffaloes indicate that soy lecithin or liposome-based extenders have a similar or better cryoprotective efficiency compared with egg-yolk based extenders. To the best of our knowledge, this is the first comparative study between liposome-, soy lecithin-, and egg yolk-based Tris extenders for cryopreservation of camel semen, and the only other study that investigated the efficiency of egg yolk-free diluents in camel sperm preservation is that of Al-Bulushi et al. [11]. Nevertheless, the investigators did not perform a post-thawing semen evaluation but only chilled the diluted semen up to 48 h. The findings of this study indicated that semen diluted in Triladyl had higher total and progressive motility, better LIN, better acrosome integrity, and similar sperm vitality at 24 h and 48 h, when compared to OPTIXcell. Our results and Al-Bulushi et al. [8] results showed that liposome- or soy lecithin-based extenders are less efficient in protecting the dromedary camel sperm cell wall and nuclear chromatin either during chilling or during deep-freezing. The effect of liposomes and soy lecithin on spermatozoa and the extent to which they alter the cholesterol/phospholipid ratio is not yet fully understood. The existing controversy in the literature regarding the efficiency of egg yolk-based replacers may be attributed to male-to-male variations in the ability of sperm to fuse with liposomes or soy lecithin because of genetic, environmental, or nutritional parameters. This variation can affect sperm plasma membrane composition and integrity [37,38]. Therefore, it is imperative to comparatively evaluate liposome- and soy lecithin-based semen extenders to routinely used egg yolk-extenders in various breeds with different environmental adaptations. 

At the pre-freezing evaluation, the only differences observed between Triladyl and SHOTOR were in regard to sperm motility kinetics (VSL, VAP, LIN, BCF). In the post-thawing evaluation, Triladyl resulted in better sperm motility and motility kinetics than SHOTOR, while SHOTOR resulted in better vitality, plasma membrane integrity, and DNA integrity. Thus, the main differences between Triladyl- and SHOTOR-diluted semen samples can be detected during freeze-thawing cycles. The sperm preservation capacity of Triladyl in dromedary camel was higher than other dromedary camel-specific (Green buffer) and bovine-specific (Biladyl) yolk-based extenders [11]. Although sperm membrane integrity, vitality, and motility are strongly associated with one another [34,39], we observed an inconsistency between sperm motility and membrane integrity. This indicates that the ability of SHOTOR to protect the motility apparatus of sperm mitochondria and axonemal apparatus is lower than its ability to protect the cell wall, and vice versa for Triladyl. Similar inconsistencies have been reported in chilled camel semen [9,40]. This may be due to the different vulnerability to cryoinjuries seen in different sperm parts [41]. Although SHOTOR resulted in significantly better post-thawing DNA integrity than Triladyl, the proportion of sperm with DNA integrity in Triladyl-diluted samples was still considered acceptable (88%). The type of penetrating cryoprotectant found to affect the cryopreservation efficacy of SHOTOR in dromedary camel with the dimethyl formamide resulted in better post-thawing sperm quality compared to glycerol, dimethyl sulphoxide, and ethylene glycol [6]. In this previous study, the post-thawing total sperm motility was about 55% after using SHOTOR, which is quite higher than that observed in the current study. Nevertheless, the glycerol concentration in El-Badry et al. [6] study was 6% while in our study it was 3%. Thus, difference between Triladyl and SHOTOR may be attributed to the different glycerol concentrations (6.4 vs. 3%, respectively), the presence of unknown constituents in Triladyl, the different types and concentrations of sugars, and/or the different main ingredients of the extenders. 

All the evaluated post-thawing sperm parameters were better in Triladyl- than Steridyl-diluted ejaculates, while the negative effect of Steridyl on sperm vitality, plasma membrane integrity, and DNA integrity was obvious even in the pre-freezing evaluation. According to published ingredients of Triladyl and Steridyl, the main difference between them is the origin of the egg yolk. The Steridyl contains irradiated sterile egg yolk while the Triladyl supplemented with in-lab prepared egg yolk. Interestingly, it has been reported that the source of plasma and egg yolk influence the ability of Tris-based extenders to maintain semen motility in chilled dromedary camel ejaculates [42]. Similarly, the egg yolk source has been shown to affect the post-thawing sperm motility and in vivo fertility in buffalo [23,43]. This could be attributed to the differences in egg yolk compositions in terms of fatty acid, phospholipids, cholesterol, and lipoprotein levels [16,44,45]. However, since the exact compositions of the commercially available semen extenders are unknown, this hypothesis should be considered with caution. Further research is required to investigate the precise cryoprotective role of soy lecithin, liposomes, and egg yolk in a defined semen extender. 

In the current study, the sperm kinematics data (VCL, VSL and VAP) for pre and post freezing considered acceptable if compared with their values recorded in fresh semen evaluated by SCA [29,30,31]. While, the sperm kinematics data evaluated by SCA and reported in current study and our previous studies are considered very low when compared with their values reported by other authors using other CASA software [7,11]. The type of CASA software may be the cause of these variations in the sperm kinematics data. Additionally, the progressive motility in the current study looks to be low when compared with their values reported by other authors [7,11] because of the progressivity cut-off value in the current study was > 80% of STR; while, it was low and reached to > 60% of STR in these other studies [7,11]. Therefore, for accurate evaluation of the progressive motility the mean of STR % must be checked.

Although we did not evaluate the ability of thawed sperm to initiate and maintain pregnancy in vivo, the CASA employed in the current study enabled the precise evaluation of numerous sperm parameters that are known to correlate with semen fertility and testosterone level [46,47,48]. In the current study Triladyl-diluted ejaculates showed better velocity, STR, LIN, ALH, and BCF, indicating that spermatozoa preserved in Triladyl have more efficient flagellar structures, ATP production, and consequently sperm tail beat frequency [34]. LIN, BCF, and ALH have been strongly correlated to fertility in dromedary camels and bulls [46,47]. Another crucial parameter that should not be neglected in semen evaluations is DNA integrity. DNA integrity has been correlated with in vitro penetration and pronuclear formation in camels [7] and fertility in bulls [47]. Triladyl resulted in lower DNA integrity than SHOTOR but still within an acceptable level.

In summary, we conclude that egg-yolk-based diluents (Tirladyl, SHOTOR) are more efficient in cryopreservation of dromedary camel semen than soys-lecithin or liposome-based extender (AndroMed and OPTIXcell). Nevertheless, the benefits of egg yolk-free extenders in terms of biosecurity should not be ignored. Further research is required to improve the cryoprotective efficiency of soy lecithin and liposomes in dromedary camel semen. Furthermore, Triladyl was the best extender compared with other extenders under investigation; further studies are needed to evaluate the AI conception rate of camels with semen diluted Triladyl. 

## 5. Conclusions

In summary, we conclude that egg-yolk based diluents (Tirladyl, SHOTOR) are more efficient in cryopreservation of dromedary camel semen than soys-lecithin or liposome-based extender (AndroMed and OPTIXcell). Nevertheless, the benefits of egg yolk-free extenders in terms of biosecurity should not be ignored. Further research is required to improve the cryoprotective efficiency of soy lecithin and liposomes in dromedary camel semen. Furthermore, Triladyl was the best extender compared with other extenders under investigation. Further studies are needed to evaluate the in vivo fertility rates of cryopreserved semen diluted by different commercial diluents.

## Figures and Tables

**Table 1:** Effect of different extenders on pre-freezing and post-thawing sperm motility and sperm motility dynamics (mean ± SE).

Time	Parameter	Semen Extenders				
		SHOTOR	Triladyl	Steridyl	OPTIXcell	AndroMed
Pre-freezing	Total motility, %	79.39 ± 1.37	81.18 ± 0.65	78.29 ± 1.34	79.91 ± 0.63	80.13 ± 0.94
	Total progressive, %	21.54 ± 1.83 ^a^	18.72 ± 0.93 ^a,b^	15.76 ± 1.80 ^b,c^	17.43 ± 1.10 ^b,c^	13.27 ± 1.07 ^c^
	VCL, µm/s	48.66 ± 0.32 ^b^	49.09 ± 0.22 ^a,b^	48.84 ± 0.29 ^b^	49.64 ± 0.22 ^a^	46.35 ± 0.27 ^c^
	VSL, µm/s	12.84 ± 0.38 ^b^	13.28 ± 0.41 ^a^	12.81 ± 0.38 ^b^	13.24 ± 0.39 ^a^	12.56 ± 0.38 ^b^
	VAP, µm/s	23.43 ± 0.16 ^b^	24.04 ± 0.12 ^a^	23.96 ± 0.15 ^a^	24.11 ± 0.12 ^a^	22.92 ± 0.14 ^c^
	LIN, %	24.98 ± 0.20 ^c^	25.98 ± 0.10 ^b^	25.11 ± 0.20 ^c^	25.82 ± 0.10 ^d^	25.58 ± 0.20 ^b^
	STR, %	50.63 ± 0.30 ^a,b^	50.87 ± 0.20 ^a^	49.95 ± 0.30 ^b^	50.80 ± 0.20 ^a^	49.15 ± 0.20 ^c^
	ALH, µm	3.43 ± 0.02 ^a^	3.34 ± 0.01 ^b^	3.41 ± 0.02 ^a^	3.43 ± 0.013 ^a^	3.19 ± 0.02 ^c^
	BCF, Hz/s	3.76 ± 0.03 ^c^	3.95 ± 0.02 ^a,b^	3.89 ± 0.02 ^b^	3.97 ± 0.02 ^a^	3.9 ± 0.02 ^a,b^
Post-thawing	Total motility, %	35.09 ± 1.341 ^b^	38.63 ± 0.81 ^a^	34.4 ± 0.84 ^b^	28.39 ± 0.86 ^c^	31.99 ± 1.48 ^b^
	Total progressive, %	5.13 ± 0.55 ^b^	10.04 ± 0.66 ^a^	5.57 ± 0.26 ^b^	3.86 ± 0.29 ^b^	3.78 ± 0.32 ^b^
	VCL, µm/s	28.98 ± 0.63 ^c^	35.25 ± 0.55 ^a^	32.56 ± 0.67 ^b^	28.03 ± 0.53 ^c^	25.65 ± 0.74 ^d^
	VSL, µm/s	8.95 ± 0.28 ^c^	13.05 ± 0.26 ^a^	10.47 ± 0.27 ^b^	8.51 ± 0.22 ^c,d^	8.02 ± 0.37 ^d^
	VAP, µm/s	14.62 ± 0.33 ^c^	17.88 ± 0.29 ^a^	16.52 ± 0.33 ^b^	14.03 ± 0.25 ^c^	12.83 ± 0.38 ^d^
	LIN, %	32.32 ± 10 ^b^	35.41 ± 0.60 ^a^	29.89 ± 10 ^c^	30.84 ± 10 ^b,c^	32.26 ± 0.70 ^b^
	STR, %	57.47 ± 10 ^b,c^	65.52 ± 0.90 ^a^	56.20 ± 10 ^c^	56.91 ± 0.80 ^b,c^	58.99 ± 0.90 ^b^
	ALH, µm	2.33 ± 0.04 ^b,c^	2.71 ± 0.03 ^a^	2.40 ± 0.04 ^b^	2.23 ± 0.03 ^c^	2.10 ± 0.05 ^d^
	BCF, Hz/s	2.84 ± 0.08 ^c^	3.87 ± 0.09 ^a^	3.76 ± 0.09 ^a^	2.92 ± 0.08 ^c^	2.91 ± 0.09 ^c^

Values with different superscripts in the same row indicate statistical significance (*p* < 0.05). VAP: average pathway velocity; VSL: straight-line velocity; VCL: curvilinear velocity; LIN: linearity coefficient; STR: straightness coefficient; ALH: amplitude of lateral head displacement; BCF: beat cross frequency.

**Table 2:** Effect of different extenders on pre-freezing and post-thawing sperm vitality, plasma membrane integrity, DNA integrity, and sperm abnormalities (mean ± SE).

Time	Parameter (%)	Semen Extenders				
		SHOTOR	Triladyl	Steridyl	OPTIXcell	AndroMed
Pre-freezing	Sperm vitality	87.22 ± 0.51 ^a^	87.41 ± 0.59 ^a^	79.11 ± 0.32 ^c^	82.42 ± 0.68 ^b^	83.65 ± 0.72 ^b^
	Plasma membrane integrity	85.48 ± 0.58 ^b^	87.77 ± 0.31 ^a^	80.00 ± 1.01 ^d^	82.00 ± 0.80 ^c^	82.30 ± 0.61 ^c^
	DNA integrity	97.33 ± 0.33 ^a^	96.00 ± 0.57 ^a^	85.33 ± 0.33 ^d^	88.88 ± 0.57 ^c^	91.00 ± 0.57 ^b^
	Sperm abnormalities	6.25 ± 0.08 ^c^	6.50 ± 0.19 ^c^	8.00 ± 0.10 ^b^	12.00 ± 0.06 ^a^	8.75 ± 0.11 ^b^
Post-thawing	Sperm vitality	57.21 ± 1.3 ^a^	51.11 ± 1.24 ^b^	46.28 ± 0.60 ^c^	38.76 ± 0.83 ^d^	39.20 ± 1.27 ^d^
	Plasma membrane integrity	59.85 ± 1.17 ^a^	52.96 ± 1.01 ^b^	45.34 ± 0.65 ^c^	37.60 ± 0.73 ^d^	38.90 ± 1.05 ^d^
	DNA integrity	91.00 ± 0.57 ^a^	88.00 ± 0.57 ^b^	83.00 ± 0.57 ^c^	73.00 ± 0.57 ^e^	78.00 ± 0.57 ^d^
	Sperm abnormalities	11.75 ± 0.081 ^c^	12.50 ± 0.10 ^c^	15.75 ± 0.11 ^b^	21.00 ± 0.077 ^a^	16.00 ± 0.08 ^b^

Values with different superscripts in the same row indicate statistical significance (*p* < 0.05).

**Table 3:** Effect of different extenders on pre-freezing and post-thawing sperm head morphometry (mean ± SE).

Time	Parameter	Semen Extenders				
		SHOTOR	Triladyl	Steridyl	OPTIXcell	AndroMed
Pre-freezing	Head area (μm^2^)	18.97 ± 0.29 ^a^	15.87 ± 0.38 ^c^	17.95 ± 0.41 ^a,b^	17.22 ± 0.51 ^b^	15.91 ± 0.34 ^c^
	Head perimeter (μm)	17.23 ± 0.19 ^a^	15.54 ± 0.37 ^c^	16.67 ± 0.26 ^a,b^	16.72 ± 0.53 ^a,b^	15.77 ± 0.38 ^b,c^
	Elongation	0.28 ± 0.01 ^a^	0.22 ± 0.01 ^b^	0.26 ± 0.01 ^a^	0.261 ± 0.01 ^a^	0.22 ± 0.01 ^b^
	Ellipticity	1.80 ± 0.04 ^a^	1.57 ± 0.04 ^b^	1.73 ± 0.04 ^a^	1.72 ± 0.04 ^a^	1.58 ± 0.04 ^b^
	Regularity	0.94 ± 0.01	0.93 ± 0.01	0.95 ± 0.01	0.94 ± 0.01	0.95 ± 0.02
	Rugosity	0.80 ± 0.01 ^a^	0.83 ± 0.02 ^a^	0.81 ± 0.01 ^a^	0.79 ± 0.02 ^b^	0.81 ± 0.02 ^a^
	Acrosome (%)	73.11 ± 2.47	68.44 ± 2.47	70.23 ± 2.36	66.25 ± 2.76	66.85 ± 2.42
Post-thawing	Head area (μm^2^)	19.04 ± 0.5	20.22 ± 0.61	19.66 ± 0.65	20.12 ± 0.73	18.75 ± 0.57
	Head perimeter (μm)	17.26 ± 0.32 ^a,b^	17.92 ± 0.34 ^a^	17.32 ± 0.34 ^a,b^	17.89 ± 0.38 ^a^	16.7 ± 0.33 ^b^
	Elongation	0.25 ± 0.01 ^a,b^	0.25 ± 0.01 ^a,b^	0.23 ± 0.01 ^b,c^	0.26 ± 0.01 ^a^	0.21 ± 0.014 ^c^
	Ellipticity	1.68 ± 0.03 ^a^	1.67 ± 0.03 ^a^	1.61 ± 0.04 ^a,b^	1.73 ± 0.03 ^a^	1.54 ± 0.04 ^b^
	Regularity	0.95 ± 0.008 ^a^	0.94 ± 0.02 ^a^	0.9 ± 0.01 ^b^	0.93 ± 0.01 ^a,b^	0.92 ± 0.01 ^a,b^
	Rugosity	0.81 ± 0.01 ^a,b^	0.79 ± 0.02 ^b^	0.82 ± 0.01 ^a,b^	0.79 ± 0.017 ^b^	0.84 ± 0.01 ^a^
	Acrosome (%)	66.89 ± 2.76	66.26 ± 3.03	68.42 ± 1.78	69.55 ± 2.41	65.47 ± 2.29

Values with different superscripts in the same row indicate statistical significance (*p* < 0.05).
